# An integrated chinmedomics strategy for discovery of effective constituents from traditional herbal medicine

**DOI:** 10.1038/srep18997

**Published:** 2016-01-11

**Authors:** Xijun Wang, Aihua Zhang, Xiaohang Zhou, Qi Liu, Yang Nan, Yu Guan, Ling Kong, Ying Han, Hui Sun, Guangli Yan

**Affiliations:** 1 National TCM Key Laboratory of Serum Pharmacochemistry, Laboratory of Metabolomics, Research Center of Chinmedomics, Department of Pharmaceutical Analysis, Heilongjiang University of Chinese Medicine, Heping Road 24, Harbin 150040, China

## Abstract

Traditional natural product discovery affords no information about compound structure or pharmacological activities until late in the discovery process, and leads to low probabilities of finding compounds with unique biological properties. By integrating serum pharmacochemistry-based screening with high-resolution metabolomics analysis, we have developed a new platform, termed chinmedomics which is capable of directly discovering the bioactive constituents. In this work, the focus is on ShenQiWan (SQW) treatment of ShenYangXu (SYX, kidney-yang deficiency syndrome) as a case study, as determined by chinmedomics. With serum pharmacochemistry, a total of 34 peaks were tentatively characterised *in vivo*, 24 of which were parent components and 10 metabolites were detected. The metabolic profiling and potential biomarkers of SYX were also investigated and 23 differential metabolites were found. 20 highly correlated components were screened by the plotting of correlation between marker metabolites and serum constituents and considered as the main active components of SQW. These compounds are imported into a database to predict the action targets: 14 importantly potential targets were found and related to aldosterone-regulated sodium reabsorption and adrenergic signaling pathways. Our study showed that integrated chinmedomics is a powerful strategy for discovery and screening of effective constituents from herbal medicines.

Herbal medicines (HM) have been widely used for their antibacterial, antifungal, anticancer, antiviral, and anti-inflammatory activities, and other pharmacological activities of benefit to humankind. Traditional Chinese medicine (TCM) has its own unique medical system with the significant characteristics of pursuing an overall therapeutic effect with a multi-target treatment^1^. It consists of multiple plants (called ‘formulae or prescription’) that could regulate balance and homeostasis of the body in a holistic fashion. The pharmacological effects of TCM formulae are demonstrated to express their effects through multiple active constituents^2^,^3^. HM are not pure products with a single active ingredient. Therefore, conventional methods for screening and identifying the active ingredients in natural products are time-consuming and labour intensive. Traditional natural product discovery, using conventional methods, affords no information about mode of action until late in the discovery process. This leads to high rates of rediscovery and low probabilities of finding compounds with unique biological properties.

Currently, HM development has been hampered due to the lack of high-throughput, rapid methods for screening and identifying bioactive constituents. Therefore, it is essential to develop a method able to overcome these limitations. Fortunately, serum pharmacochemistry has been shown to elucidate the *in vivo* constituents and metabolites of TCM^4^: because only compounds in the blood have the probability of becoming effective constituents, the serum pharmaco-chemistry of TCM (SPT) can reveal drug action, absorption, distribution, and interaction in the body. In recent years, the LC-MS analytical method has been successfully applied to TCM formulae^5^,^6^,^7^. From the perspective of modern medicine, disease is a functional state caused by the body’s metabolic imbalances. Metabolomics can capture comprehensive analysis of small molecule metabolites and provides a powerful approach to establish metabolic profiling, to discover metabolite biomarkers and related disease pathways^8^. To determine which constituents contributed to the therapeutic effects, correlation analysis was performed and the correlation coefficient (*r*) described the degree of correlation between the metabolite biomarkers and chemical composition *in vivo*. Correlation analysis between metabolite markers with serum constituents originated from TCM is used for discovery of the bioactive constituents. Recently, by integrating SPT-based screening with high-resolution metabolomics analysis, we have developed a new platform, termed chinmedomics which is capable of direct discovery and screening of highly correlated components with the therapeutic effect of TCM^9^. The complex relationships between the chemical composition *in vivo* and the efficacy of TCM have also been taken into account.

With the development of TCM, many preparations such as granules, capsules, pills, and injections are now produced to cater to the demands of customers. TCM has played an indispensable role in the prevention and treatment of diseases in most Asian countries^10^. TCM considers the kidney is the most important system in the body, and considers that “kidney yang” motivates the power of human vitality^11^. ShenYangXu (SYX, kidney-yang deficiency syndrome) is a complex kidney disorder^12^,^13^. Chen *et al.* had used GC/MS metabolomics to investigate *in vivo* urine biochemical modification of kidney deficiency syndromes induced by high doses of hydrocortisone. 23 endogenous urinary metabolites of rats perturbed after treatment with hydrocortisone were measured by GC/MS, and these substances are involved in multiple biochemical processes, such as energy metabolism, lipid metabolism, and amino acid metabolism. Their work suggested that metabolomics could be used as a powerful tool to investigate the metabolic mechanisms of kidney deficiency syndromes^14^. ShenQiWan (SQW) is a typical TCM formula for invigorating SYX and has been so for thousands of years in Asia: it was first recorded in the “Synopsis of the Golden Chamber”, consisting of *Radix Rehmanniae Preparata, Fructus Macrocarpii, Rhizoma Dioscoreae Oppositae, Rhizoma Alismatis, Poria, Cortex Moutan Radicis, Radix aconiti lateralis preparata,* and *Ramulus Cinnamomi.* Nevertheless, due to its complex constituents, it is not easy to explore the mechanism of action of SQW using traditional methods, and little is known about the change in bioactive constituents in SYX treated with SQW. In this study, we have selected SQW as a case study for bioactive constituent analysis through use of a chinmedomics strategy. The objective of this study was to develop an effective strategy for screening and identifying effective substances in HM, and to address most of the aforementioned challenges inherent in studying complex HM.

## Experimental Work

### Chemicals and materials

Methanol (HPLC grade) was purchased from Fisher Scientific Corporation (Loughborough, UK); acetonitrile, HPLC grade, was obtained from Merck (Darmstadt, Germany); leucine enkephalin was purchased from Sigma-Aldrich (St Louis, MO, USA). The deionised water (18.2 MΩ) was further purified using a Milli-Q system (Millipore, Billerica, USA); formic acid and phosphoric acid was of an analytical grade purchased from Beijing Reagent Company (Beijing, China). *Radix Rehmanniae Preparata, Fructus Macrocarpii, Rhizoma Dioscoreae Oppositae, Rhizoma Alismatis, Poria, Cortex Moutan Radicis, Radix aconiti lateralis preparata,* and *Ramulus Cinnamomi* were obtained from Harbin Shiyitang Drug Store (Harbin, China), and authenticated by Prof. Xijun Wang. Voucher specimens were deposited at the authors’ laboratory. Commercial SQW were purchased from Shiyitang Co., Ltd (Harbin, China).

### Preparation of SQW samples

According to the original composition and preparation method of SQW recorded in the ‘Synopsis of the Golden Chamber’, the SQW was prepared as follows: *Radix Rehmanniae Preparata, Fructus Macrocarpii, Rhizoma Dioscoreae Oppositae, Rhizoma Alismatis, Poria, Cortex Moutan Radicis, Radix aconiti lateralis preparata,* and *Ramulus Cinnamomi* were mixed in proportions 8:4:4:3:3:3:1:1, and then ground, mixed, and then reflux extracted in a rotary evaporator with six times the volume of 100% methanol for 2 h (twice), then the filtrate was freeze-dried. The freeze-dried powder was dissolved in water to make 1.08 g/ml solutions.

### Animal handling

Male Wistar rats (body mass 200 to 240 g) were supplied by the GLP Centre, Heilongjiang University of Chinese Medicine (Harbin, China). The room temperature was regulated to 21 ± 5 °C with 60 ± 5% relative humidity. A 12 h light-dark cycle was set and there was free access to standard diet and water. The raw SQW powder was dissolved in distilled water to form a stock solution (0.3389 g/ml). This solution was orally administrated to male Wistar rats (1 ml per 100 g body mass). The animals were allowed to acclimatise in metabolism cages for 7 days before dosing and were put in metabolism cages during the experimental period. After acclimatisation, the animals were randomly divided into three groups with fifteen rats in each group: the control, model, and SQW groups. The rats of the control group were firstly induced by i.p. injection of olive oil for 21 consecutive days. The rats in the model group were induced by i.p. injection of corticosterone at a dose of 10 mg/kg per day for 21 consecutive days. The SQW group was treated in the same way as the model group during the first 21 days. After that the SQW group was orally administrated an accurate volume of solution of SQW for 15 consecutive days. The experimental protocols were approved by the Animal Care and Use Committee of Heilongjiang University of Chinese Medicine (HUCM-2014-08206). The experimental methods were conducted according to the principles expressed in the Declaration of Helsinki.

### Sample collection and preparation

Blood was collected from the abdominal aorta, and plasma and serum were separated by centrifugation at 4000 rpm for 10 mins at 4 °C after standing for 30 mins. The supernatant was collected in micro-tubes and stored at −80 °C before analysis. The levels of biochemical assay followed manufacturer instructions on commercial kits (data not shown). All procedures complied with manufacturer guidelines. The serum samples were thawed before analysis. Proteins were precipitated from the defrosted serum samples (200 μL) by adding four volumes of methanol in 1.5 mL micro-tubes at room temperature. After vortexing for 10 s, supernatants (800 μL) were collected after centrifugation at 13,000 rpm for 10 minutes. Supernatants were dried under nitrogen and redissolved with 80% methanol to volumes of 200 μL. The mixture solution was filtered through a 0.22 mm PTFE membrane, and transferred to vials for UPLC/MS analysis.

### Metabolomics study

#### UPLC analysis

Chromatography was performed on an UPLC system (Waters Corp., USA) with an autosampler at 4 °C: separation was carried out on an HSS T_3_ column (100 mm × 2.1 mm i.d., 1.8 μm, Waters Corporation, USA). The column temperature was maintained at 40 °C. The analysis was performed with gradient elution using (A) acetonitrile with 0.1% formic acid, and (B) water with 0.1% formic acid as the mobile phase: the flow rate was set to 0.4 mL/min. The gradient eluting conditions were: 0 to 2 min, linear increase from 5% to 50% A; 2 to 4 min, 50% to 60% A; 4 to 7 min, 60% to 80% A; 7 to 10 min, 80%to 100% A; 10 to 12 min, maintain 100% A; 12 to 12.5 min, linear decrease from 100% to 5% A; held at 5% A for 2 min for equilibration of the column. The sample injection volume was 3 μl. The QC samples were used to optimise the condition of UPLC/MS, as it contained the most information of all plasma samples. Throughout the analysis, all samples were maintained at 4 °C.

#### MS analysis

A Waters Acquity™ Mass Spectrometer (Waters Corp., Milford, USA) was connected to the UPLC system via an electrospray ionisation (ESI) interface. The ESI source was operated in positive ionisation mode with the capillary voltage at 3.0 kV, sample cone voltage at 30 V, and extraction cone voltage at 4 V. The temperatures at the source and desolvation ends were set to 110 °C and 450 °C, respectively. The cone and desolvation gas flow rates were maintained at 50 L/h and 500 L/h, respectively. In negative ion mode, the sample cone voltage was 40 V, and the desolvation temperature was 300 °C. The data acquisition rate was set to 0.3 s/scan, with a 0.1 s (0.02 interscan time) interscan delay. Data were collected in centroid mode from 50 to 2000 Da. For accurate mass acquisition, a lock-mass of leucine enkephalin was used to ensure accuracy during the MS analysis.

#### Metabolite analysis

MS data acquired were performed to Markerlynx within Masslynx software (Version 4.1) for peak detection and alignment. The multivariate data matrix was input to EZinfo Ver. 2.0 software (Waters Corp., Milford, USA) for principal component analysis (PCA), partial least-squares-discriminant analysis (PLS-DA), and orthogonal projection to latent structures (OPLS) analysis. The molecular ion distribution was found by mass spectrometry and subsequently, the molecular weight was calculated. The MassFragment™ application manager (Waters Corp., Milford, USA) was used to facilitate the MS/MS fragment ion analysis process by way of chemically intelligent peak-matching algorithms.

### Constituent analysis *in vitro* and *in vivo*

#### Sample preparation for constituent analysis

The methanol extraction powder of SQW was centrifuged at 13,000 rpm for 15 minutes at 4 °C, and the supernatant was filtered through a 0.20 μm filter: the 5 μl filtrate was used as UPLC sample. Phosphoric acid (40 μL) was added to 2 ml of the serum supernatant and ultrasonicated for 1 minute, and vortexed for 30 s. The mixed solution was applied to a pre-activated OASIS HLB solid phase extraction C_18_ column (30 μm, 60 mg, Waters Corp., USA). The column was washed with 1 ml of water and 2 mL of 100% methanol. The 100% methanol eluates were collected and dried under nitrogen gas at 45 °C. The residues were redissolved in 150 μL of methanol and centrifuged at 13,000 rpm for 15 minutes at 4 °C, filtered through a 0.20 μm filter: the 5 μL filtrate was used as the UPLC sample.

### Instrumentation and conditions

Chromatographic separation was performed with an Acquity™ UPLC BEH C_18_ column (100 mm × 2.1 mm i.d., 1.7 μm particle size, Waters Corp., Milford, MA, USA) maintained at 35 °C. A gradient with eluent A (HCOOH to H_2_O ratio of 1:1000, v/v) and B (HCOOH to CH_3_CN of 1:1000, v/v) was used at a flow rate of 0.5 ml/min. The linear elution gradient program was used as follows: 0 to 2.0 min, 90 to 65% A; 2.0 to 4.0 min, 65 to 40% A; 4.0 to 8.0 min, 40 to 20% A; 8.0 to 8.5 min, 20 to 1% A; 8.5 to 10.5 min, 1% A; 10.5 to 11.5 min, 1 to 90% A; 11.5 to 13.0 min, 90% A. The sample-tray temperature was kept at 4 °C.

The MS instrument consisted of a Waters Synapt^TM^ QTOF/ HDMS (Waters Corp., USA). Ionisation was performed in both positive and negative ion modes. The MS source temperature was set to 110 °C, and the desolvation temperature was set to 300 °C with a desolvation gas flow of 500 L/h. The capillary voltage was 4 kV. The mass spectrometric data were collected in full scan mode across the range of *m*/*z* 100 to 1000 Da, with accurate mass measurement of all mass peaks. The collision energy was set to 10 to 30 eV for low-energy scans, and 30 to 50 eV for high-energy scans. MetaboLynx^TM^ (Waters Corp., USA) was used when analysing the chemical constituents and *in vivo* metabolites in TCM formulae. Metabolynx^TM^ is capable of automatically processing LC/MS data sets to search for endogenous and exogenous metabolites.

### Correlation analysis of marker metabolites and absorbed constituents

A plot of the correlation model between the marker metabolites and chemical composition (PCMS) was used to screen the constituents or metabolites absorbed into the blood after oral administration of SQW. To determine which constituents contributed most to the therapeutic effect, the correlation coefficient (*r*) described the degree of correlation between two variables: all the markers and the chemical composition of the SQW were assayed *in vivo*. In this study, we extracted the relevant chemical component data when *r* ≥ 0.7 and identified the individual components using PCMS.

### Potential target prediction of highly correlated ingredients

In this study, we extracted and identified the relevant chemical components absorbed into blood when *r* ≥ 0.7. The selected compounds are imported into the Herbal Ingredients’ Target database (HIT, http://lifecenter.sgst.cn/hit/) to predict the biological targets, and then the target numbers were entered into the KEGG database (http://www.genome.jp/kegg/) to annotate and analyse the pathway.

## Results

### Metabolomic analysis of SQW on SYX syndrome

#### Multivariate statistical analysis

**Figure 1:**
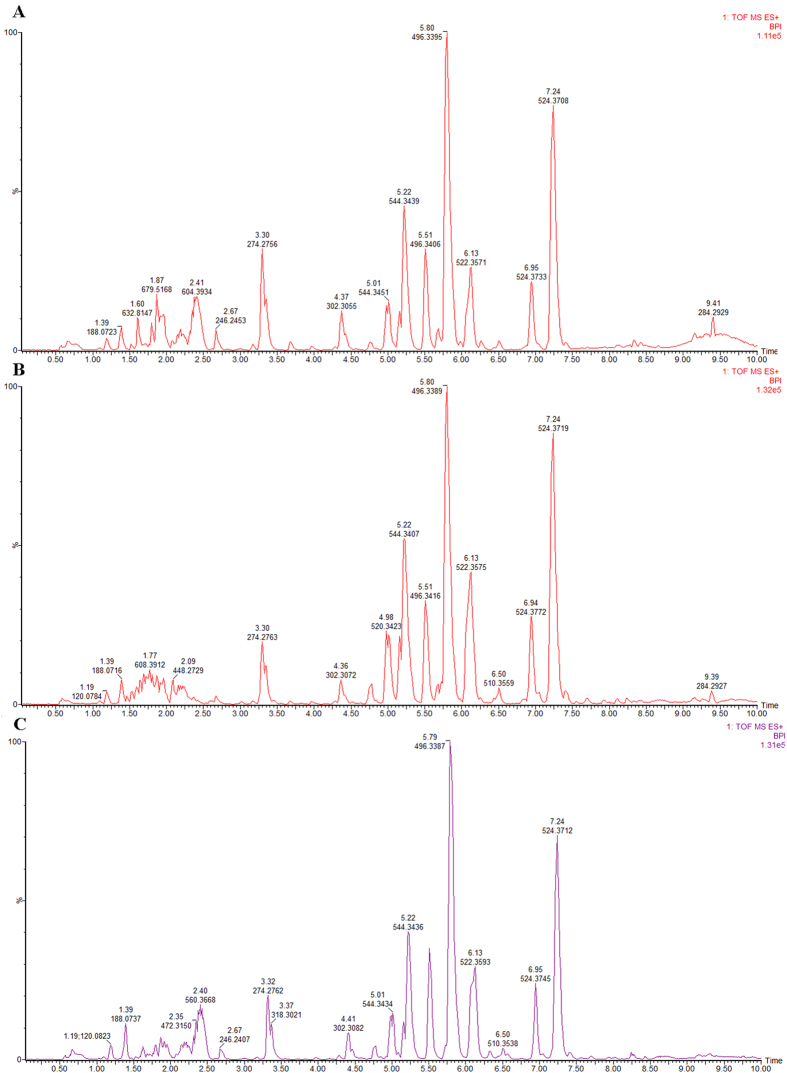
Base peak ion chromatograms of plasma samples. (**A**) control group, (**B**) model group, and (**C**) SQW group.

**Figure 2:**
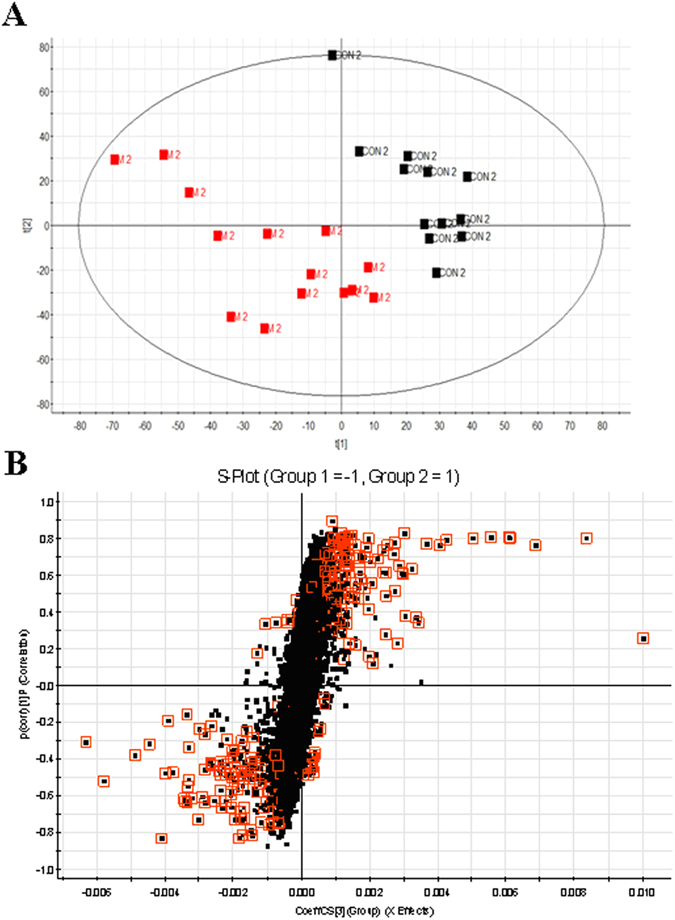
Multivariate data analyses of the UPLC/MS serum spectra data. (**A**), Score plots of PCA based on serum metabolite profiling (■, control group; 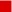, model group); (**B**), The S-plot of PLS-DA for the control and model groups.

The typical base peak intensity (BPI) chromatograms of the biosamples that were collected from representative rats from each group are presented in Fig. 1. Raw data from UPLC/MS were analysed by the MarkerLynx software. Multivariate data analysis was performed using EZinfo 2.0 software, and there was an obvious separation between the control and model groups, suggesting that biochemical perturbation happened in the SYX group (Fig. 2A). The S-plot of OPLS was drawn to find the metabolic biomarkers of SYX (Fig. 2B). The furthest metabolite ions from the origin of the S-plot of OPLS exhibiting a higher value were potential biomarkers, and were responsible for the difference between control and model groups.

#### Identification of endogenous metabolites

**Figure 3:**
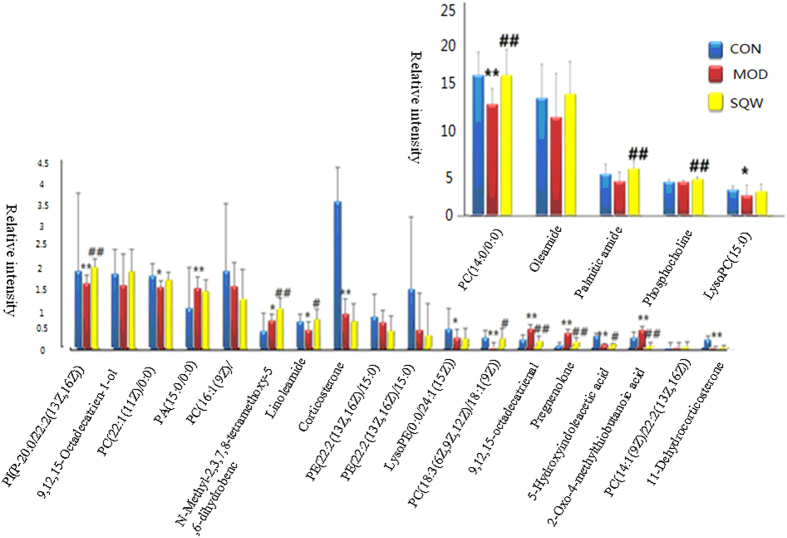
Relative signal intensities of serum metabolites identified by UPLC/MS.

Molecular mass was determined within a reasonable degree of measurement error (<5 ppm) using Q-TOF/MS, and the potential element composition, and degree of saturation of the compounds were obtained. To identify these metabolites, these variables were predicted by comparing the accurate MS and MS/MS fragments with the metabolites found when searching on-line databases. According to the protocol described above, a total of 23 ions (*p* < 0.05) contributed to the classification of the control and model groups (Table S1). Among them, five of the 23 biomarkers identified were up-regulated and 18 of them were down-regulated in the SYX group shown in Fig. 3.

#### Protective effects of SQW in SYX syndrome

**Figure 4:**
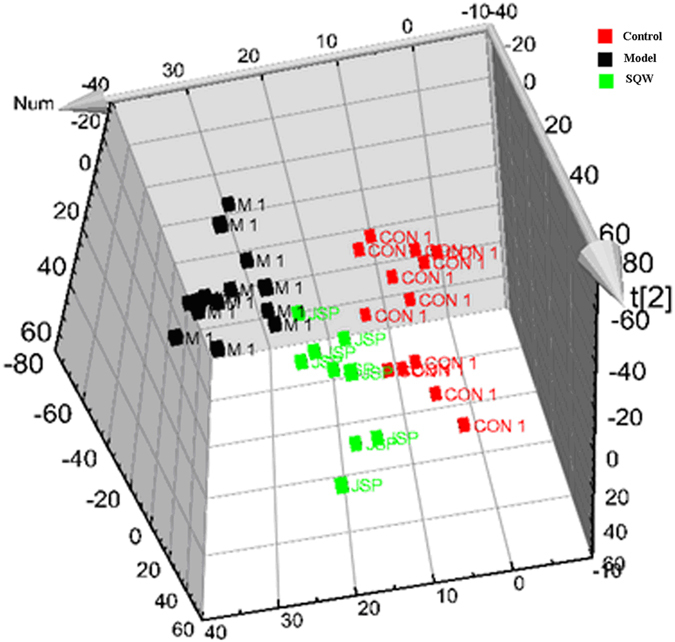
The 3-d PCA plot for SQW treatment on SYX (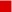, control group; ■, model group;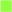, SQW group).

From the 3-d score plot of the PCA, a clear separation among the control, model, and SQW groups was seen in Fig. 4. The score plot of PCA showed that the control, and model groups were separated clearly, and SQW treatment group was much closer to the control group than the model group, which suggested that SQW could restore the pathological process of SYX. To determine whether SQW could influence the metabolic pattern induced by SYX, the intensities of the metabolites were compared among the control, SYX, and SQW groups. Of note, 17 of the identified biomarkers were completely reversed by SQW (Fig. 3). To determine whether our observations of changes in the 17 metabolites reflected the changes in metabolic pathways, we used bioinformatics analysis MetPA software to identify the network pathway related to the metabolic mechanisms of SQW in SYX syndrome. These selected metabolites involved glycerophospholipid metabolism, steroid hormone biosynthesis, tryptophan metabolism, and cysteine and methionine metabolism, suggesting that these changes may be in response to therapeutic effects for the SQW in SYX syndrome.

### Constituents analysis of SQW

#### Rapid discovery and global characterisation of multiple constituents from SQW

**Figure 5:**
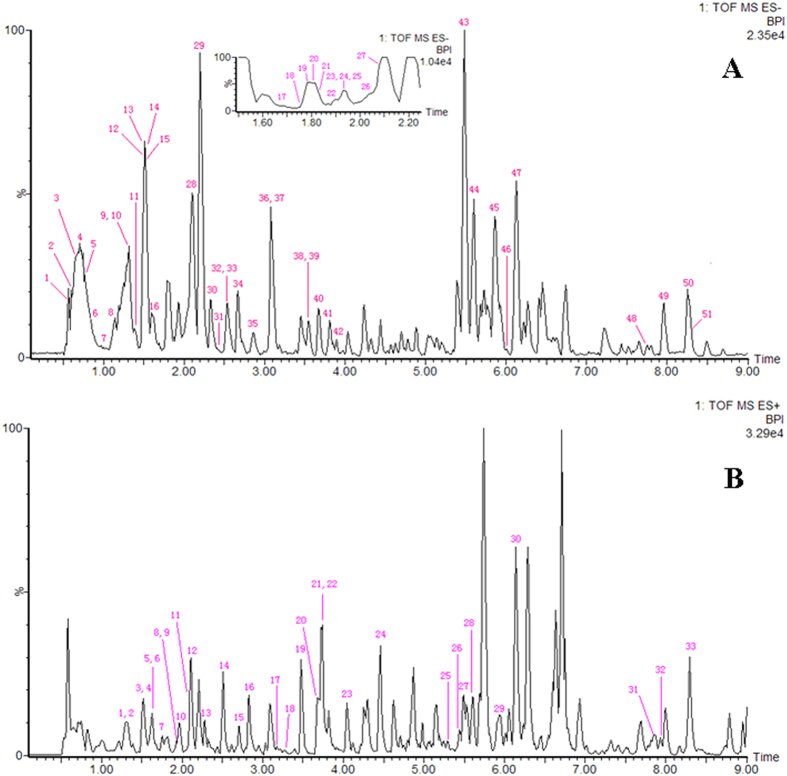
ESI base peak ion chromatogram of the SQW analysed by UPLC/QTOF MS in negative ion mode (A) and positive ion mode (B).

**Figure 6:**
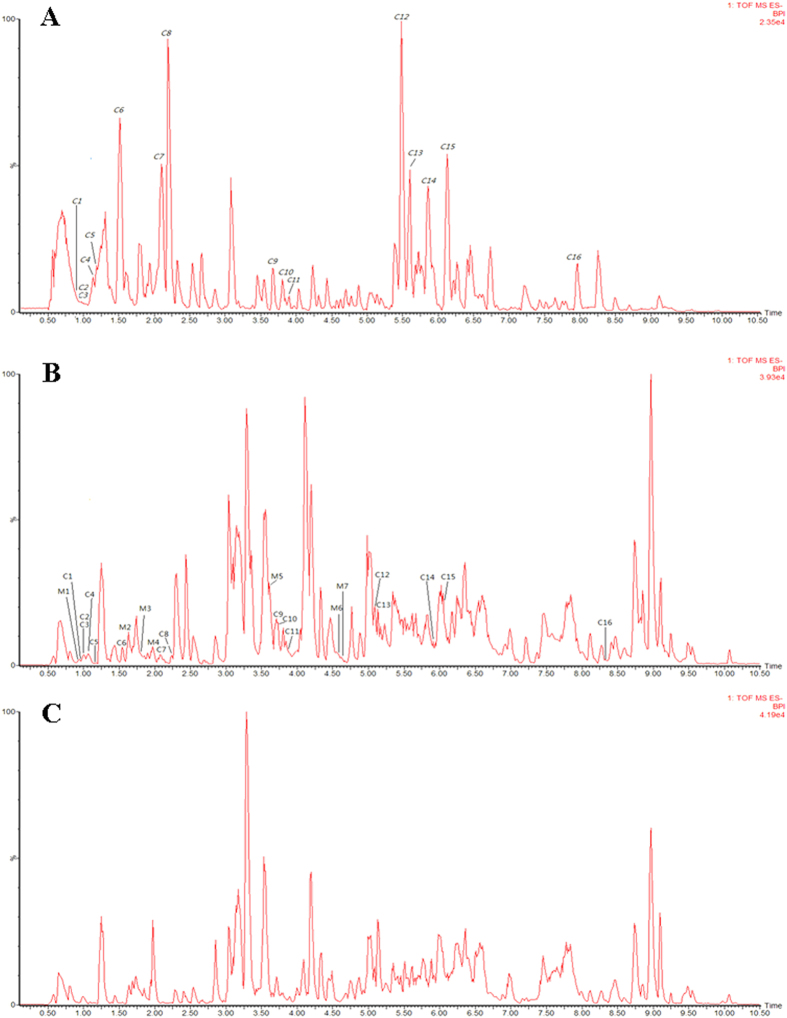
UPLC-HDMS chromatograms of SQW in negative ion mode. The peak numbers are listed in Table S2. (**A**), *in vitro*; (**B**), dosed serum; (**C**), control serum.

**Figure 7:**
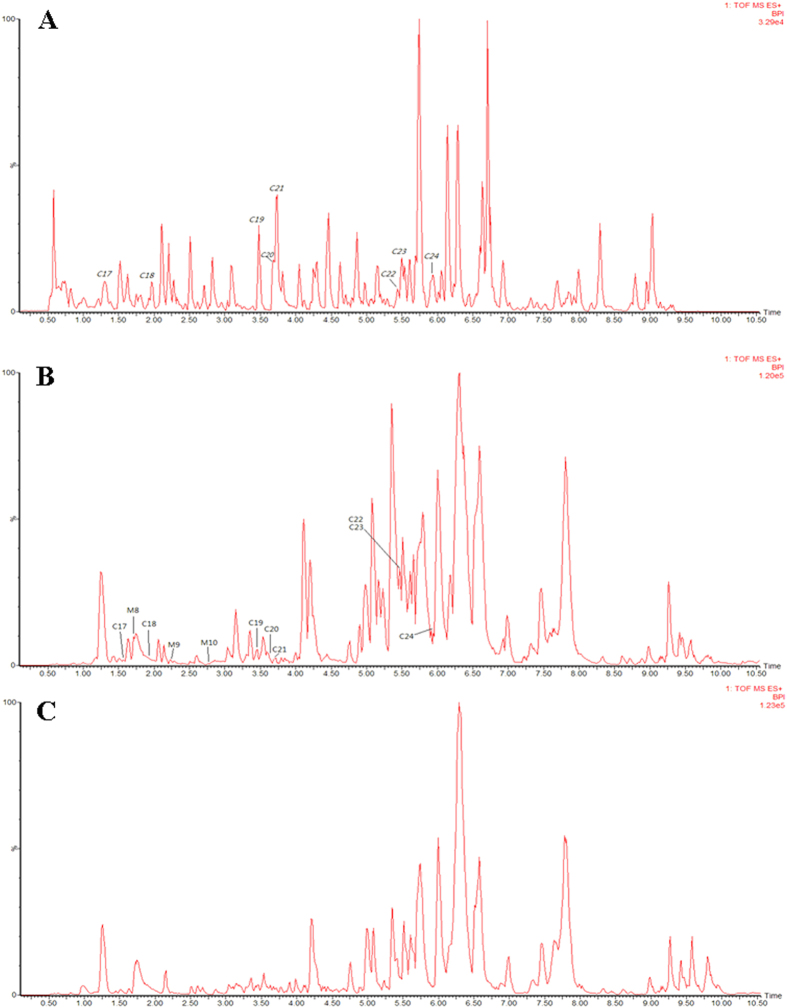
UPLC-HDMS chromatograms of SQW in positive ion mode. The peak numbers are listed in Table S3. (**A**), *in vitro*; (**B**), dosed serum; (**C**), control serum.

UPLC/Q-TOF-MS/MS with automated MS^E^ (E represents collision energy) data analysis software (MetaboLynx^TM^) was used to analyse and identify the chemical components of SQW. The high- and low-energy MS^E^ tool generates product ion spectra which were successfully used in the structural elucidation of detected SQW components. The chemical formula of an unknown structure was deduced based on high-accuracy [M−H]^−^/[M + HCOO]^−^ (in negative ion mode) or [M + H]^+^/[M + Na]^+^ (in positive ion mode) precursor ions. As a result, a total of 84 compounds, were identified, or tentatively characterised, from the constituents of SQW. The ESI BPI chromatogram of the SQW by UPLC/QTOF MS is shown in Fig. 5. By UPLC/Q-TOF-MS/MS analysis, 51 compounds (Fig. 6 and Table S2) in negative ion mode and 33 compounds (Fig. 7 and Table S3) in positive ion mode were identified from SQW.

### *In vivo* analysis of SQW using Metabolynx™

**Figure 8:**
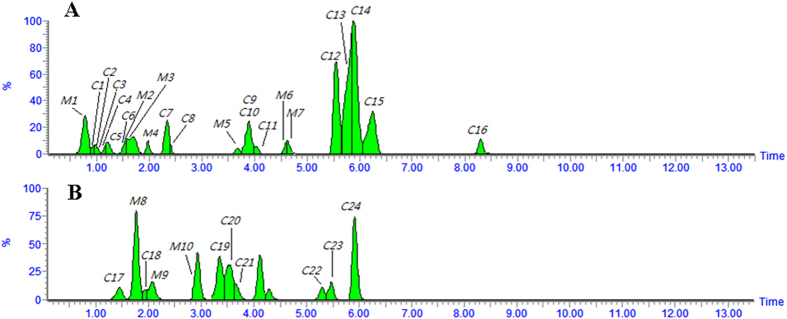
Extracted ions chromatogram of rat serum after oral administration of SQW with Metabolynx™ in negative ion mode (A) and positive ion mode (B).The peak numbers are listed in Table S4 and S5.

Metabolynx™ software was used to screen the absorbed constituents and metabolites in analytes, compared with control samples. When a peak was included as a metabolite, the peak area in the analyte had to be at least three times greater than that of the control. The bioactive compounds in SQW could be discovered by comparative analysis of the chemical profiles of a control sample and a dosed sample: they were then identified based on their MS and MS/MS. The extracted ion chromatogram of plasma samples with the Metabolynx™ tool are presented in Fig. 8, and the products were well separated using the developed UPLC method. Analysis of the peaks found nine metabolites (Table S4 and Table S5) which indicated that glucuronide conjugation, decarbonylation, and deethylation were the major metabolic pathways of constituents *in vivo*. To illustrate the dynamic profiles of absorbed constituents and metabolites *in vivo*, further research would be required into the relevant pharmacokinetics to understand the absorption, distribution, and excretion of these components.

### Correlation analysis between marker metabolites and absorbed constituents

**Figure 9:**
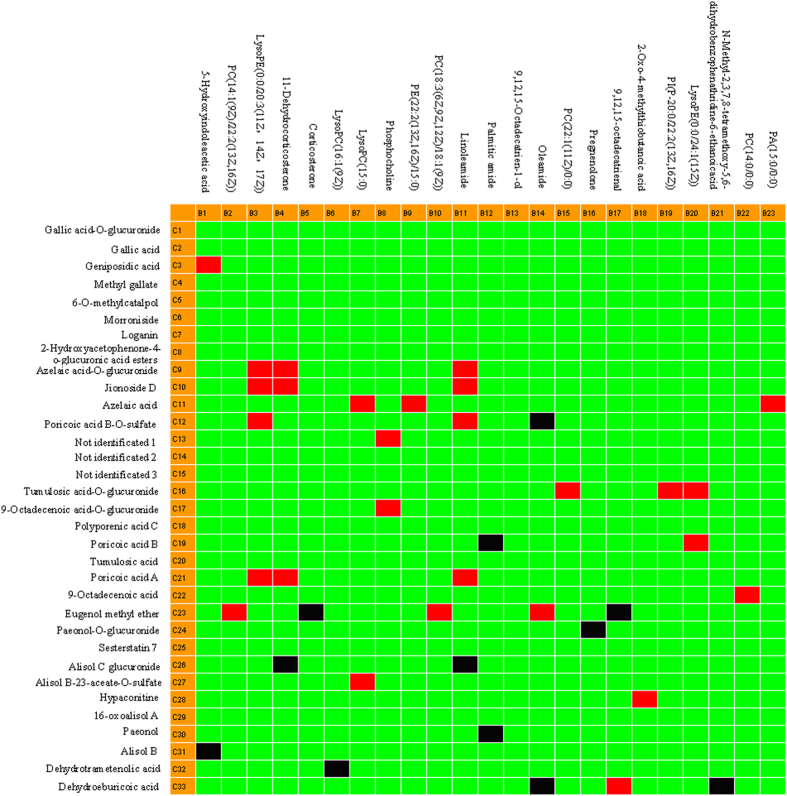
Correlation between marker metabolites and serum constituents in SQW. Note: 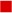, highly positively correlated; ■, highly negatively correlated; 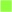, low correlation; left column, chemical components; top column, marker metabolites. The correlation coefficients are listed in Table S6.

A correlation model between the marker metabolites and their chemical composition was established, under the premise of therapeutic efficacy, to discover the constituents or metabolites absorbed into the blood after oral administration of SQW. To determine which constituents contributed to the mainly therapeutic effect of SQW against SYX syndrome, we extracted the relevant data when *r* ≥ 0.7 using PCMS software. The absorbed constituents that were highly correlated with the therapeutic effect of SQW are shown in Fig. 9. According to correlation coefficient (Table S6), 20 absorbed components such as azelaic acid-O-glucuronide, jionoside D, azelaic acid, *etc.*, were highly positively (*r* ≥ 0.7) and negatively correlated (*r* ≤ −0.7) with protective effects of SQW in SYX syndrome. Therefore, we hypothesised that these components might play important roles in the therapeutic effect of SQW. Interestingly, azelaic acid-O-glucuronide, jionoside D, azelaic acid, poricoic acid B-O-sulfate, tumulosic acid-O-glucuronide, poricoic acid A, eugenol methyl ether, and dehydroeburicoic acid had an extremely strong relationship with the therapeutic effect of SQW against SYX syndrome. Trials are on-going to find the activity of these compounds.

### Potential target prediction of the correlated ingredients

Based on our study, 20 compounds absorbed into the blood were highly correlated with the therapeutic effect of SQW in SYX syndrome, were selected to predict the biological targets. The 20 compounds were imported into the Herbal Ingredients’ Target database (http://lifecenter.sgst.cn/hit/) to predict the targets. Results showed that 14 potential protein targets (prediction score ≥0.6) were found (Table S7), such as mineralocorticoid receptor, glucocorticoid receptor, alpha-1 adrenergic receptor, tyrosinase, phenylalanine-4-hydroxylase, arachidonate 5-lipoxygenase, prolyl 3-hydroxylase 2, *etc.* These targets were put into the KEGG pathway annotation: two pathways including aldosterone-regulated sodium reabsorption and adrenergic signalling were discovered.

## Discussion

Phytomedicine is a part of health care systems around the world: the World Health Organisation estimates that 80% of the global population rely on herbs for their primary health care needs^15^,^16^. TCM is one of the oldest phytomedical health care systems, and it has been used in Asian countries such as China, Japan, and Korea for thousands of years. Based on its long history of clinical use and sound effects in the treatment of numerous diseases, especially chronic diseases, TCM is widely accepted and used by billions of people around the world. For improving health, practitioners advocated combinatorial therapeutic strategies based on overall symptoms and signs of syndromes and often prescribe a combination of herbs called formulae that work together to achieve their therapeutic effects^17^. A syndrome is a basic description of the disease in TCM, and formulae are corresponding drugs against a syndrome. A single herb already contains thousands of compounds, however formulae consisting of several herbs form system of different chemical compositions. It maybe causes many difficulties in the discovery of the bioactive constituents thereof: in the early 1990s, we first established SPT, providing a methodology for the discovery of active constituents *in vivo* from TCM. Metabolomics, monitoring the entire pattern of low molecular weight compounds rather than focusing on individual metabolites, suits TCM, thus it is an effective approach in the investigation of how each formula works^18^,^19^,^20^,^21^,^22^. In the 21st century, SPT was integrated with metabolomics, to develop a system called c*hinmedomics* for elucidating the bioactive constituents from TCM. The overall procedure of the chinmedomic analysis is shown in Figure S1. Briefly, metabolomic technology is used to clarify possible metabolic mechanisms of a syndrome, and SPT is used to discover the active compounds in the body after oral TCM; under the premise of the effectiveness of formulae, correlation analysis between the exogenous compounds and endogenous marker metabolites *in vivo* are used to clarify the bioactive constituents^23^.

In this work, we give an illustrative example to show that the effective substances of TCM can be found by the proposed chinmedomics method. On the basis of the biological characterisation of a syndrome, we will establish metabolic profiling and fingerprints of animal models of SYX syndrome, and evaluate the overall effects of TCM formulae and the corresponding relationship between syndrome and formulae. In this study, we explored serum metabolomic profiling and potential metabolic markers as well as an analysis of its chemical constituents to reveal the treatment mechanism of SQW in SYX rats. A total of 23 biomarkers, contributing to the classification of the control and model groups, were identified. SQW presented protective effects by reversing 17 potential biomarkers to control levels. Using the potential biomarkers found in this study as an index, we found that SQW had obvious recovery effects on SYX through reversing partially disturbed metabolic pathways. MetaboLynx™ provides an efficient approach in reducing matrix ions from biological samples, the screening of metabolites in complex biological matrices, and discrimination of endogenous and exogenous metabolites. It indicated that 24 components in the SQW were absorbed into rat body tissue and nine metabolites *in vivo* were identified by UPLC/Q-TOF-MS/MS coupled with the Metabolynx™ tool. A fingerprinting method applied to SPT was used to identify the chemical constituents of SQW *in vivo*: 84 compounds in SQW and 33 compounds *in vivo* were identified by the SPT method. Under the premise of therapeutic efficacy, an optimal plot of the correlation model between the marker metabolites and chemical composition (PCMS using Pearson’s correlation) was used to screen the effective substances after oral administration of SQW formulae. According to the correlation coefficient, the absorbed components that were positively highly, and negatively, correlated to marker metabolites, contributed most of the therapeutic effect. By correlation analysis of these compounds *in vivo* and marker metabolites, 20 components were identified as drug candidates. Based on our study, 20 compounds absorbed into blood were highly correlated with therapeutic effect of SQW in SYX syndrome, were selected to predict the biological targets. It showed that 14 potential targets were found which were related to aldosterone-regulated sodium reabsorption and adrenergic signalling pathways.

As a hotspot of TCM studies, chinmedomics has gradually drawn attention from those seeking to clarify the effective substances of TCM. There are a variety of methods used to establish the chemical fingerprint and the serum fingerprint of TCM. To overcome the shortcomings of serum fingerprints, it uses a data-processing method to analyse the weight coefficients of each component. The processing methods used are not uniform, and include: correlation analysis, cluster analysis, regression analysis, pattern recognition analysis, *etc*. Through the analytic statistics method, chinmedomics could establish a mathematical model of TCM’s chemical composition *in vivo*, its efficacy, and active compounds. Moreover, it established a relationship between the absorbed fingerprints and their bioactivity to identify important components by correlation analysis, which could provide evidence of effective constituents from the TCM formulae. Being an interdisciplinary science, the proposed method is a technology integrating analytical chemistry, SPT, metabolomics, and chemometrics, and is felt to be more accurate when evaluating the multiple components, and multi-target properties, of TCM.

In summary, the establishment and implementation of chinmedomics aimed to explore the intrinsic link between the essence of syndrome and formulae efficacy, as well as potentially bioactive constituents. SQW bestows a protective effect by regulating the perturbed marker metabolites from their normal states; this helped to understand better the therapeutic effect of SQW. Under the premise of therapeutic efficacy, a total of 33 peaks were tentatively characterised *in vivo* by the use of UPLC/QTOF MS coupled with automated detection by Metabolynx™ tool, 24 of which were parent components, with nine metabolites being detected: this provided helpful chemical information for further pharmacological research. PCMS analysis showed that 20 compositions that had a relationship with therapeutic effect, and might play important roles in the therapeutic effect of SQW. The activity of these compounds needed verify by the subsequent assay. In this work, it was concluded that the multiple components of SQW caused a therapeutic effect on SYX syndrome. This strategy could increase knowledge of the bioactive constituents from TCM in a high-throughput manner.

## Additional Information

**How to cite this article**: Wang, X. *et al.* An integrated chinmedomics strategy for discovery of effective constituents from traditional herbal medicine. *Sci. Rep.* **6**, 18997; doi: 10.1038/srep18997 (2016).

## Supplementary Material

Supplementary Information
