# Supplementary material for: An integrated chinmedomics strategy for discovery of effective constituents from traditional herbal medicine
Source: Sci Rep. 2016 Jan 11;6:18997. doi: 10.1038/srep18997 (PMC4707445; doi:10.1038/srep18997)
Supplement: Supplementary Information [file srep18997-s1.pdf]

# An integrated chinmedomics strategy for discovery of effective constituents from traditional herbal medicine

Xijun Wang[‡] *, Aihua Zhang[‡], Xiaohang Zhou, Qi Liu, Yang Nan, Yu Guan, Ling Kong, Ying Han, Hui Sun, Guangli Yan

*National TCM Key Laboratory of Serum Pharmacochemistry, Laboratory of Metabolomics, Research Center of Chinmedomics, Department of Pharmaceutical Analysis, Heilongjiang University of Chinese Medicine, Heping Road 24, Harbin 150040, China*

Address correspondence to:

Prof. Xijun Wang

National TCM Key Laboratory of Serum Pharmacochemistry

Laboratory of Metabolomics

Research Center of Chinmedomics

Department of Pharmaceutical Analysis

Heilongjiang University of Chinese Medicine

Heping Road 24

Harbin 150040, China

Tel. & Fax +86-451-82110818

Email: xijunwangls@126.com, aihuatcm@163.com

[‡] These authors contributed equally to this work.

**Table S1.** Identification of marker metabolites related to SYX based on serum metabolic profiling.

| NO. | Rt | m/z determined | m/z calculated | Error (ppm) | Ion form | Formula | Metabolite Name |
|---|---|---|---|---|---|---|---|
| 1 | 1.2 | 192.0665 | 192.0661 | 2.1 | [M+H]+ | $C_{10}H_9NO_3$ | 5-Hydroxyindoleacetic acid |
| 2 | 1.69 | 806.5677 | 806.5676 | 0.1 | [M+Na]+ | $C_{44}H_{82}NO_8P$ | PC(14:1(9Z)/22:2(13Z,16Z)) |
| 3 | 2.4 | 504.3106 | 504.309 | 3.2 | [M+H]+ | $C_{25}H_{46}NO_7P$ | LysoPE(0:0/20:3(11Z，14Z，17Z)) |
| 4 | 2.62 | 345.2073 | 345.2066 | 2 | [M+H]+ | $C_{21}H_{28}O_4$ | 11-Dehydrocorticosterone |
| 5 | 2.72 | 347.2227 | 347.2222 | 1.4 | [M+H]+ | $C_{21}H_{30}O_4$ | Corticosterone |
| 6 | 4.79 | 494.3204 | 494.3247 | -8.7 | [M+H]+ | $C_{24}H_{48}NO_7P$ | LysoPC(16:1(9Z)) |
| 7 | 4.81 | 482.3244 | 482.3247 | -0.6 | [M+H]+ | $C_{23}H_{48}NO_7P$ | LysoPC(15:0) |
| 8 | 5.82 | 184.0744 | 184.0739 | 2.7 | [M+H]+ | $C_5H_{14}NO_4P$ | Phosphocholine |
| 9 | 6.12 | 758.5714 | 758.57 | 1.8 | [M+H]+ | $C_{42}H_{80}NO_8P$ | PE(22:2(13Z,16Z)/15:0) |
| 10 | 6.12 | 782.5699 | 782.57 | -0.1 | [M+H]+ | $C_{44}H_{80}NO_8P$ | PC(18:3(6Z,9Z,12Z)/18:1(9Z)) |
| 11 | 7.29 | 280.2658 | 280.264 | 6.4 | [M+H]+ | $C_{18}H_{33}NO$ | Linoleamide |
| 12 | 8 | 256.2646 | 256.464 | 2.3 | [M+H]+ | $C_{16}H_{33}NO$ | Palmitic amide |
| 13 | 8.25 | 265.2558 | 265.2531 | 10.2 | [M+H]+ | $C_{18}H_{32}O$ | 9,12,15-Octadecatrien-1-ol |
| 14 | 8.26 | 282.2799 | 282.2797 | 0.7 | [M+H]+ | $C_{18}H_{35}NO$ | Oleamide |
| 15 | 8.71 | 578.4189 | 578.4186 | 0.5 | [M+H]+ | $C_{30}H_{60}NO_7P$ | PC(22:1(11Z)/0:0) |
| 16 | 9 | 317.2502 | 317.2481 | 6.6 | [M+H]+ | $C_{21}H_{32}O_2$ | Pregnenolone |
| 17 | 9.89 | 263.2384 | 263.2375 | 3.4 | [M+H]+ | $C_{18}H_{30}O$ | 9,12,15-octadecatrienal |
| 18 | 10.53 | 149.0243 | 149.0227 | -19.5 | [M+H]+ | $C_5H_8O_3S$ | 2-Oxo-4-methylthiobutanoic acid |
| 19 | 5.5 | 975.6534 | 975.6538 | -0.3 | [M+FA-H]- | $C_{51}H_{95}O_{12}P$ | PI(P-20:0/22:2(13Z,16Z)) |
| 20 | 8.69 | 562.387 | 562.3903 | -5.9 | [M-H]- | $C_{29}H_{58}NO_7P$ | LysoPE(0:0/24:1(15Z)) |
| 21 | 10.15 | 468.1658 | 468.1658 | 0 | [M+FA-H]- | $C_{24}H_{25}NO_6$ | N-Methyl-2,3,7,8-tetramethoxy-5,6-dihydrobenzophenathridine-6-ethanoicacid |
| 22 | 5.08 | 466.2918 | 466.2934 | -3.4 | [M-H]- | $C_{22}H_{46}NO_7P$ | PC(14:0/0:0) |
| 23 | 8 | 395.2193 | 395.2199 | -8.1 | [M-H]- | $C_{18}H_{37}O_7P$ | PA(15:0/0:0) |

**Table S2.** Characterisation and chemical structure of constituents in SQW (ESI-).

| NO. | Retention time | Identified compounds | [M-H] | Actual_M | Element composition | MS/MS（Losses） | ppm | Origin |
|---|---|---|---|---|---|---|---|---|
| 1 | 0.55 | Verbascose | 827.2666 | 828.2747 | C30H52O26 | 665[-C6H10O5]<br>485[-C12H22O11]<br>179[-C24H40O20] | -0.4 | a |
| 2 | 0.56 | Stachyose | 665.2144 | 666.2219 | C24H42O21 | 503[-C6H10O5]<br>341[-C12H20O10]<br>179[-C18H30O15]<br>161[-C18H32O16] | 0.6 | a |
| 3 | 0.69 | Tartaric acid | 149.0092 | 150.0164 | C4H6O6 | 103[-CH2O2]<br>87[-CH2O3]<br>72[-C2H4O3]<br>59[-C2H2O4] | 4.0 | b |
| 4 | 0.70 | Malic acid | 133.0140 | 134.0215 | C4H6O5 | 115[-H2O]<br>89[-CO2]<br>71[-CH2O3]<br>59[-C2H2O3] | 2.3 | b |
| 5 | 0.84 | Catalpol | 361.1732 | 362.1213 | C15H22O10 | 271[-C4H10O2]<br>211[-C6H14O4]<br>169[-C8H16O5]<br>125[-C9H16O7]<br>111[-C10H18O7] | 高 | a |
| 6 | 0.89 | Gallic acid | 169.0138 | 170.0215 | C7H6O5 | 135[-H2O2]<br>125[-CO2]<br>107[-H2O-COOH] | 0.6 | b,e |
| 7 | 0.93 | Geniposidic acid | 373.1140 | 374.1213 | C16H22O10 | 747[2M-H]<br>211[-C6H10O5]<br>191[-C6H14O6]<br>179[-C10H10O4] | 1.3 | i |
| 8 | 1.14 | 6-O-methylcatalpol | 375.1292 | 376.1370 | C16H24O10 | 285[-C3H6O3]<br>271[-C5H12O2]<br>243[-C5H8O4]<br>213[-C6H10O5]<br>169[-C9H18O5]<br>125[-C10H18O7]<br>59[-C14H20O8] | 0.3 | a |

| | | | | | | | | |
|---|---|---|---|---|---|---|---|---|
| 9 | 1.30 | Morroniside | 405.1390 | 406.1475 | C17H26O11 | 361[-C2H4O]<br>243[-C6H10O5]<br>155[-C10H18O7]<br>141[-C10H16O8]<br>123[-C10H18O9]<br>101[-C13H20O8]<br>95[-C12H22O9] | -1.7 | b |
| 10 | 1.30 | Oxypaeoniflorin | 495.1509 | 496.1581 | C23H28O12 | 465[-CH2O]<br>333[-C6H10O5]<br>137[-C16H22O9]<br>93[-C17H22O11] | 1.2 | e |
| 11 | 1.40 | (+)-Catechin | 289.0723 | 290.0790 | C15H14O6 | 245[-C2H4O]<br>221[-C3O2]<br>161[-C6H8O3]<br>151[-C6H2O4]<br>137[-C8H8O3]<br>125[-C9H8O3]<br>109[-C9H8O4] | 3.8 | e |
| 12 | 1.50 | Echinacoside | 785.2499 | 786.2582 | C35H46O20 | 623[-C6H10O5]<br>301[-C23H32O11]<br>275[-C21H34O14]<br>179[-C29H34O14]<br>161[-C29H36O15]<br>133[-C30H36O16] | -0.6 | a |
| 13 | 1.51 | Loganin | 389.1450 | 390.1526 | C17H26O10 | 779[2M-H]<br>435[+HCOOH]<br>227[−C6H10O5] | 0.5 | b |
| 14 | 1.52 | Methyl gallate | 183.0302 | 184.0372 | C8H8O5 | 169[-CH2]<br>151[CH4O]<br>124[-CH4O] | 4.9 | e |
| 15 | 1.52 | Paeonolide | 459.1494 | 460.1581 | C20H28O12 | 293[-C9H10O3]<br>165[-C11H18O9]<br>150[-C12H21O9] | -2.0 | e |
| 16 | 1.60 | Apiopaeonoside | 459.1495 | 460.1581 | C20H28O12 | 293[-C9H10O3]<br>233[C11H14O5 ]<br>165[-C11H18O9]<br>150[-C12H21O9] | -1.7 | e |
| 17 | 1.68 | Jionoside A1<br>or<br>Jionoside A2 | 799.2669 | 800.2739 | C36H48O20 | 623[-C10H8O3]<br>295[-C22H32O13]<br>163[-C30H36O15]<br>149[-C30H34O16]<br>170[-C30H36O17] | 1.0 | a |

| | | | | | | | | |
|---|---|---|---|---|---|---|---|---|
| 18 | 1.75 | 1,2,3,6-tetro-O-galloyl-glucose | 787.0994 | 788 | C34H28O22 | 635[-C7H6O4]<br>617[-C7H8O5]<br>483[-C13H4O9]<br>465[-C14H12O9]<br>447[-C14H14O10]<br>295[-C21H18O14]<br>277[-C21H18O15]<br>169[-C27H24O17]<br>125[-C28H22O19] | -0.4 | b,e |
| 19 | 1.78 | Dihydromelittoside | 525.1819 | 526.1898 | C21H34O15 | 479[-C2H6O]<br>449[-C3H8O2]<br>327[-C6H14O7]<br>165[-C12H24O12]<br>121[-C14H28O13] | -0.4 | a |
| 20 | 1.81 | Paeoniflorin | 479.1551 | 480.1632 | C23H28O11 | 449[-CH2O]<br>327[-C5H12O5]<br>267[-C11H16O4]<br>165[-C14H18O8]<br>121[-C16H22O9] | -0.4 | e |
| 21 | 1.83 | Suffruticoside A<br>Or<br>Suffruticoside C | 611.1605 | 612.1690 | C27H32O16 | 445[-C9H10O3]<br>301[-C15H18O7]<br>169[-C20H26O11]<br>165[-C18H22O13]<br>125[-C21H26O13] | -1.1 | e |
| 22 | 1.87 | Suffruticoside B<br>or<br>Suffruticoside D | 611.1626 | 612.1690 | C27H32O16 | 445[-C9H10O3]<br>301[-C15H18O7]<br>169[-C20H26O11]<br>165[-C18H22O13]<br>125[-C21H26O13] | 2.3 | e |
| 23 | 1.93 | Forsythiaside<br>Or<br>Acteoside | 623.1970 | 624.2054 | C29H36O15 | 461[-C9H6O3]<br>161[-C20H30O12]<br>133[-C21H30O13] | -0.6 | a |
| 24 | 1.93 | Jionoside B1<br>Or<br>Jionoside B2 | 813.2820 | 814.2896 | C37H50O20 | 637[-C10H8O3]<br>301[-C21H36O14]<br>283[-C21H38O15]<br>191[-C30H38O14]<br>175[-C27H42O17]<br>161[-C31H40O15] | 0.4 | a |
| 25 | 1.93 | 1,2,3,4,6-Pentagalloylglucose | 939.1124 | 940.1182 | C41H32O26 | 769[-C7H6O5]<br>617[-C14H10O9]<br>301[C28H14O18]<br>169[-C14H10O9]<br>125[-C35H26O23] | 2.1 | e |
| 26 | 2.05 | Galloyl-paeoniflorin | 631.1161 | 632.1741 | C30H32O15 | 613[-H2O]<br>491[C7H8O3] | -0.3 | e |

| | | | | | | | | |
|---|---|---|---|---|---|---|---|---|
| | | | | | | 463[-C8H8O4] | | |
| | | | | | | 313[-C17H18O6] | | |
| | | | | | | 271[-C19H20O7] | | |
| | | | | | | 211[-C21H24O9] | | |
| | | | | | | 169[-C23H26O10] | | |
| | | | | | | 125[-C24H26O12] | | |
| 27 | 2.06 | Forsythiaside | 623.1976 | 624.2054 | C29H36O15 | 461[-C9H6O3] | 0 | a |
| | | Or | | | | 161[-C20H30O12] | | |
| | | Acteoside | | | | 133[-C21H30O13] | | |
| 28 | 2.13 | Oxybenzoyloxypaeoniflorin | 615.1705 | 616.1145 | C30H32O14 | 585[-CH2O] | | e |
| | | | | | | 477[-C7H6O2] | | |
| | | | | | | 447[-C8H8O4] | | |
| | | | | | | 281[-C14H22O8] | | |
| | | | | | | 239[-C19H20O8] | | |
| | | | | | | 165[-C20H18O12] | | |
| | | | | | | 137[C23H26O10] | | |
| | | | | | | 121[-C23H25O11] | | |
| 29 | 2.19 | Cornuside | 541.1543 | 542.1636 | C24H30O14 | 379[-C6H10O5] | 2.6 | b |
| | | | | | | 347[-C7H14O6] | | |
| | | | | | | 169[-C17H24O9] | | |
| | | | | | | 125[-C18H24O11] | | |
| 30 | 2.28 | Jionoside D | 637.2122 | 638.2211 | C30H38O15 | 475[-C6H10O5] | -1.6 | a |
| | | | | | | 461[-C9H4O4] | | |
| | | | | | | 295[-C15H18O9] | | |
| | | | | | | 175[-C24H30O9] | | |
| | | | | | | 169[-C21H24O12] | | |
| | | | | | | 161[-C21H32O12] | | |
| | | | | | | 133[-C24H24O12] | | |
| | | | | | | 125[-C24H32O12] | | |
| 31 | 2.41 | Azelaic acid | 187.0981 | 188.1049 | C9H16O4 | 169[-H2O] | 5.9 | f |
| | | | | | | 125[-H2O-COO] | | |
| 32 | 2.51 | Martynoside | 651.2299 | 652.2367 | C31H40O15 | 505[-C6H10O4] | 1.5 | a |
| | | | | | | 487[-C6H12O5] | | |
| | | | | | | 475[-C10H8O3] | | |
| | | | | | | 459[-C10H8O4] | | |
| | | | | | | 337[-C15H22O7] | | |
| | | | | | | 265[-C17H22O10] | | |
| | | | | | | 193[-C21H30O11] | | |
| | | | | | | 175[-C21H32O12] | | |
| | | | | | | 160[-C22H35O12] | | |
| 33 | 2.54 | Mudanpioside C | 599.1760 | 600.1843 | C30H32O12 | 581[-H2O] | -0.8 | e |
| | | | | | | 551[-H2O-CH2O] | | |
| | | | | | | 447[-C8H8O3] | | |
| | | | | | | 431[-C8H8O4] | | |
| | | | | | | 281[-C14H22O8] | | |

| | | | | | | | | |
|---|---|---|---|---|---|---|---|---|
| | | | | | | 239[-C19H20O7]<br>179[-C21H24O9]<br>149[-C25H22O8]<br>137[-C23H26O10] | | |
| 34 | 2.67 | Benzoylpaeoniflorin | 599.1743 | 600.1843 | C30H32O12 | 569[-CH2O]<br>477[-C7H6O2]<br>403[-C11H16O3]<br>281[-C14H22O8]<br>149[-C21H22O11]<br>137[C23H26O10]<br>121[-C23H25O11] | -3.7 | e |
| 35 | 2.76 | Cinnamic acid | 147.0462 | 148.0524 | C9H8O2 | 117[-O2]<br>91[-C2O2]<br>59[-C7H4] | 4.9 | f |
| 36 | 3.08 | Benzoylpaeoniflorin | 583.1816 | 584.1894 | C30H32O12 | 553[-CH2O]<br>519[-O4]<br>431[-C9H12O2]<br>121[-C23H26O10] | -0.3 | e |
| 37 | 3.09 | Mudanpioside B | 629.1866 | 630.1949 | C31H34O14 | 599[-CH2O]<br>477[-C8H8O3]<br>461[-C8H8O4]<br>431[-C9H10O5]<br>167[-C23H26O10]<br>137[-C24H28O11]<br>121[-C24H28O12] | 0.6 | e |
| 38 | 3.52 | Achybidensaponin Ⅰ | 955.4637 | 956.4981 | C48H76O19 | 910[-COOH]<br>835[-C4H8O4]<br>793[-C6H10O5] | -2.8 | h |
| 39 | 3.56 | Achybidensaponin Ⅱ | 793.4389 | 794.4453 | C42H66O14 | 731[-H2O-CO2]<br>631[-C6H10O5]<br>569[-C6H10O5-H2O-CO2] | 1.9 | h |
| 40 | 3.72 | Not identificated | 295.0974 | 296.1048 | C18H16O4 | 277[-H2O]<br>233[-H2O-COO]<br>205[-H2O-COO-CO] | 1.4 | f |
| 41 | 3.78 | Not identificated | 295.0967 | 296.1048 | C18H16O4 | 277[-H2O]<br>233[-H2O-COO]<br>205[-H2O-COO-CO] | -1.0 | f |
| 42 | 3.90 | Not identificated | 295.0972 | 296.1048 | C18H16O4 | 277[-H2O]<br>233[-H2O-COO]<br>205[-H2O-COO-CO] | 0.7 | f |
| 43 | 5.55 | Polyporenic acid C | 481.3311 | 482.3396 | C31H46O4 | 437[-CO2]<br>311[-C9H14O3]<br>153[-C22H32O2] | -1.5 | c |
| 44 | 5.66 | Poricoic acid B | 483.3123 | 484.3189 | C30H44O5 | 453[-O2]<br>437[-CH2O2]<br>390[-C6H5O]<br>337[-C7H14O3] | 2.7 | c |

| | | | | | | | | |
|---|---|---|---|---|---|---|---|---|
| 45 | 5.92 | Tumulosic acid | 485.3633 | 486.3710 | C31H50O4 | 191[-C19H32O2]<br>126[-C22H29O4]<br>469[-CH4]<br>467[-H2O]<br>423[-CH2O3]<br>337[-C6H12O4] | 0.4 | c |
| 46 | 6.01 | Neoalisol | 487.3423 | 488.3500 | C30H48O5 | 399[-C4H8O2]<br>381[-C4H10O3]<br>277[-C12H18O3] | 0 | d |
| 47 | 6.07 | Poricoic acid A | 497.3269 | 498.3345 | C31H46O5 | 453[-C3H8]<br>435[-CH2O3]<br>397[-C5H8O2]<br>351[-C7H14O3]<br>147[-C22H38O3] | 0.4 | c |
| 48 | 7.76 | Alisol B monoacetate | 513.3578 | 514.3658 | C32H50O5 | 451[-C3H10O]<br>295[-C13H14O3]<br>146[-C23H42O3] | -0.4 | d |
| 49 | 7.97 | Dehydropachymic acid | 525.3568 | 526.3658 | C33H50O5 | 465[-CO3]<br>448[-C2H5O3]<br>432[-C2H5O4]<br>421[-C3H4O4]<br>355[-C10H18O2]<br>315[-C12H18O3] | -2.3 | c |
| 50 | 8.25 | Pachymic acid | 527.3734 | 528.3815 | C33H52O5 | 465[-C2H6O2]<br>405[-C8H10O]<br>211[-C18H25O4]<br>193[-C19H25O5] | -0.4 | c |
| 51 | 8.34 | 9-Octadecenoic acid | 281.2484 | 282.2560 | C18H34O2 | 255[-C2H6]<br>227[-C2H6-CO] | 1.1 | f |

Note: a: Rehmanniae Radix; b:Cornus officinalis;c:Wolfiporia cocos;d:Alisma orientalis Juzep;e:Moutan Cortex;f:Cinnamomum cassia Presl; g:Aconitum carmichaeli;h:Achyranthese bidentata;i:Semen Plantaginis

**Table S3**. Characterisation and chemical structure of constituents in SQW (ESI+).

| NO. | Retention time | Identified compounds | [M+H] | Actual_M | Element composition | MS/MS | ppm | Origin |
|---|---|---|---|---|---|---|---|---|
| 1 | 1.30 | 7-dehydrologanin | 389.1456 | 388.1369 | C17H24O10 | 551[+C6H10O5]<br>227[-C6H10O5]<br>179[-C7H14O7]<br>151[-C8H14O8] | 2.1 | b |
| 2 | 1.30 | Dehydromorroniaglycone | 227.0916 | 226.0841 | C11H14O5 | 209[-H2O]<br>195[-CH3OH]<br>177[-CH3OH-H2O]<br>149[-CH3OH-HCOOH]<br>123[-CH3OH-3H2O]<br>109[-H2O-C4H7COOH] | -1.3 | b |
| 3 | 1.51 | Loganin | 391.1610 | 390.1526 | C17H26O10 | 229[-C6H10O5]<br>213[-C6H10O6]<br>197[-C7H14O6]<br>179[-C11H16O4]<br>167[-C8H16O7]<br>161[C11H18O5]<br>151[-C8H16O8] | 1.5 | b |
| 4 | 1.52 | Eugenol methyl ether | 179.0717 | 178.0994 | C11H14O2 | 151[-C2H4]<br>137[-C3H6]<br>123[-C4H8]<br>109[-C5H10] | | b |
| 5 | 1.63 | Sweroside | 359.1350 | 358.1264 | C16H22O9 | 197[-C6H10O5 ]<br>179[-C6H12O6]<br>151[-C11H12O4]<br>127[-C10H16O6]<br>111[-C9H12O8] | 2.2 | b |
| 6 | 1.63 | 5-hydroxymethylfurfural | 127.0397 | 126.0317 | C6H6O3 | 109[-H2O]<br>97[-CH2O] | 1.6 | b |
| 7 | 1.77 | Talatisamine | 422.2912 | 421.2828 | C24H39NO5 | 390[-CH4O]<br>358[-C2H8O2]<br>298[-C5H16O3] | 1.4 | g |
| 8 | 1.93 | Sesterstatin 7 | 445.2965 | 444.2876 | C27H40O5 | 429 [-CH4]<br>329 [-C6H12O2]<br>372 [-C4H9O]<br>301 [-C8H16O2]<br>241 [-C15H24]<br>117 [-C21H43O2] | 2.5 | h |
| 9 | 1.94 | Verbascoside | 625.2170 | 624.2054 | C29H36O15 | 479[-C6H10O4]<br>471[-C8H10O3]<br>325[-C14H20O7]<br>163[-C20H30O12] | 6.1 | i |
| 10 | 1.96 | Plantagoside | 467.1209 | 466.1111 | C21H22O12 | 305[-C6H10O5]<br>179[-C15H12O6]<br>163[-C15H12O7]<br>145[-C15H14O8] | 4.1 | i |

| | | | | | | | | |
|---|---|---|---|---|---|---|---|---|
| 11 | 2.06 | Isoverbascoside | 625.2177 | 624.2054 | C29H36O15 | 479[-C6H10O4]<br>471[-C8H10O3]<br>325[-C14H20O7]<br>163[-C20H30O12] | 7.2 | i |
| 12 | 2.12 | 1-[4,6-Di(1-piperidinyl)-1,3,5-triazin-2-yl]hydrazinecarboxamide | 321.2173 | 320.2073 | C14H24N8O | 207 [-C3H21N4]<br>199 [-C4HN4O]<br>170 [-C9H15N2]<br>163 [-C9H22N2]<br>149 [-C9H22N3]<br>101 [-C12H18N3O]<br>79 [-C12H26N4O] | 6.8 | h |
| 13 | 2.28 | 1,1'-[1,10-Decanediylbis(oxy)]bis(6,6-dimethyl-1,6-dihydro-1,3,5-triazine-2,4-diamine) | 453.3409 | 452.3340 | C20H40N10O2 | 396 [-C3H7N]<br>340 [-C3H7N5]<br>282 [-C6H13N5O]<br>226 [-C8H17N7O]<br>149 [-C14H36N6O] | -1.1 | h |
| 14 | 2.51 | Benzoylmesaconine | 590.2993 | 589.2890 | C31H43NO10 | 572[-H2O]<br>558[-CH4O]<br>540[-CH6O2]<br>526[-C2H8O2]<br>508[-C2H10O3]<br>482[-C4H12O3] | 4.7 | g |
| 15 | 2.7 | Benzoylaconine | 604.3115 | 603.3043 | C32H45NO10 | 586[-H2O]<br>572[-CH4O]<br>554[-CH6O]<br>522[-C2H10O3]<br>508[-C3H12O3] | -1.2 | g |
| 16 | 2.82 | Benzoylhypaconine | 574.3039 | 573.2938 | C31H43NO9 | 542[-CH4O]<br>510[-C2H8O2] | 4.0 | g |
| 17 | 3.19 | Mesacontine | 632.3069 | 631.2993 | C33H45NO11 | 572[-C2H4O2] | -0.3 | g |
| 18 | 3.23 | Aconifine | 662.3210 | 661.3098 | C34H47NO12 | 602[-C2H4O2]<br>542[-C7H4O2]<br>105[-C27H43NO11] | 5.0 | g |
| 19 | 3.48 | Hypaconitine | 616.3122 | 615.3043 | C33H45NO10 | 584[-CH4O]<br>556[-C2H4O2]<br>524[-C3H8O3]<br>492[-C4H12O4]<br>338[-C16H24NO3] | 4.2 | g |
| 20 | 3.68 | 16-oxoalisol A | 505.3535 | 504.3451 | C30H48O6 | 487[-H2O]<br>469[-2H2O]<br>451[-3H2O]<br>415[-C4H10O2]<br>397[-C4H12O3] | 1.2 | d |
| 21 | 3.74 | Paeonol | 167.0718 | 166.0629 | C9H10O3 | 149[-C2H6]<br>121[-C2H6O] | 6.0 | e |
| 22 | 3.75 | Deoxyaconitine | 630.3301 | 629.3200 | C34H47NO10 | 570[-C2H4O2]<br>538[-C3H8O3]<br>510[-C4H8O4] | 3.6 | g |

| | | | | | | | | |
|---|---|---|---|---|---|---|---|---|
| 23 | 4.05 | Pachymic acid | 529.3546 | 528.3815 | C33H52O5 | 469[-C3H8O]<br>451[-C3H10O2]<br>415[-C7H14O]<br>299[-C13H26O3] | -4.3 | c |
| 24 | 4.47 | Alisol C | 487.3440 | 486.3354 | C30H46O5 | 469[-H2O]<br>451[-2H2O]<br>397[-H2O-C4H8O]<br>353[-H2O-C6H12O2] | 3.5 | d |
| 25 | 5.29 | Alisol C 23-acceate | 529.3528 | 528.3451 | C32H48O6 | 511[-H2O]<br>469[-C2H4O2]<br>451[-C3H10O2]<br>433[-C5H4O2]<br>157[C23H32O4] | -0.2 | d |
| 26 | 5.49 | Alisol B | 473.3624 | 472.3552 | C30H48O4 | 455[-H2O]<br>437-[2H2O]<br>383[-H2O-C4H8O]<br>365[-2H2O-C4H8O] | -1.5 | d |
| 27 | 5.50 | Dehydrotrametenolic acid | 455.3433 | 454.3447 | C30H46O3 | 437[-OH] | 3.0 | c |
| 28 | 5.61 | 25-anhydroalisol A 11-acetate | 515.3737 | 514.3658 | C32H50O5 | 497[-H2O]<br>437[-H2O-C2H4O2]<br>419[-2H2O-C2H4O2]<br>383[-H2O-C6H10O2]<br>339[-H2O-C8H14O3]<br>213[-C16H30O5]<br>173[-C19H34O5] | 0.3 | d |
| 29 | 5.94 | Dehydroeburicoic acid | 469.3645 | 468.3603 | C31H48O3 | 451[-H2O]<br>313[-C9H16O2] | -7.9 | c |
| 30 | 6.14 | 25-anhydroalisol A 24-acetate | 515.3745 | 514.3658 | C32H50O5 | 497[-H2O]<br>437[-H2O-C2H4O2]<br>419[-2H2O-C2H4O2]<br>383[-H2O-C6H10O2]<br>339[-H2O-C8H14O3]<br>213[-C16H30O5]<br>173[-C19H34O5] | 1.7 | d |
| 31 | 7.87 | 11-deoxyalisol B-23-aceate | 499.3796 | 498.3709 | C32H50O4 | 425[-C4H10O]<br>299[-C12H24O2]<br>273[-C15H30O] | 1.6 | d |
| 32 | 7.94 | AlisolB-23-aceate | 515.3745 | 514.3658 | C32H50O5 | 497[-H2O]<br>437[-H2O-C2H4O2]<br>419[-2H2O-C2H4O2]<br>365[-H2O-C4H8O-C2H4O2]<br>339[-H2O-C8H14O3] | 1.7 | d |
| 33 | 8.21 | 11-deoxyalisol C | 471.3479 | 470.3396 | C30H46O4 | 453[-H2O]<br>435[-2H2O]<br>381[-H2O-C4H8O] | 1.1 | d |

Note: a: Rehmanniae Radix; b:Cornus officinalis;c:Wolfiporia cocos;d:Alisma orientalis Juzep;e:Moutan Cortex;f:Cinnamomum cassia Presl;g:Aconitum carmichaeli;h:Achyranthese bidentata;i:Semen Plantaginis

**Table S4**. Identified results of the constituents in rat plasma after oral administration of SQW in negative ion mode.

| NO. | Rt | Identified compounds | [M-H] | mv(Da) | Formula | MS/MS data |
|---|---|---|---|---|---|---|
| M1 | 0.88* | Gallic acid-O-glucuronide | 345.0444 | 346.0536 | $C_{13}H_{14}O_{11}$ | 169[M-(GluA-H2O)],135[-H2O2], 125[-CO2] |
| C1 | 0.89 | Gallic acid | 169.0138 | 170.0215 | $C_7H_6O_5$ | 135[-H2O2],125[-CO2] |
| C2 | 0.93 | Geniposidic acid | 373.1140 | 374.1213 | $C_{16}H_{22}O_{10}$ | 747[2M-H],211[-C6H10O5] 191[-C6H14O6],179[-C10H10O4] |
| C3 | 0.98 | Methyl gallate | 359.0659 | 360.0693 | $C_8H_8O_5$ | 169[-CH2],151[CH4O] 124[-CH4O] |
| C4 | 1.14 | 6-O-methylcatalpol | 375.1292 | 376.1370 | $C_{16}H_{24}O_{10}$ | 285[-C3H6O3],213[-C6H10O5] 169[-C9H18O5] |
| C5 | 1.30 | Morroniside | 405.1390 | 406.1475 | $C_{17}H_{26}O_{11}$ | 361[-C2H4O],243[-C6H10O5] 155[-C10H18O7],141[-C10H16O8] |
| C6 | 1.51 | Loganin | 389.1450 | 390.1526 | $C_{17}H_{26}O_{10}$ | 227[−C6H10O5] |
| M2 | 1.64* | 2-Hydroxyacetophenone-4-o-glucuronic acid esters | 327.0716 | 328.0794 | C14H16O9 | 151[-GluA-H2O] |
| M3 | 1.94* | Azelaic acid-O-glucuronide | 363.1344 | 364.1369 | $C_{15}H_{24}O_1$ | 187[-(GluA-H2O)], 175[(GluA-H2O)-H]- |
| C7 | 2.28 | Jionoside D | 637.2122 | 638.2211 | $C_{30}H_{38}O_{15}$ | 461[-C9H4O4]，295[-C15H18O9] 169[-C21H24O12],161[-C21H32O12] |
| C8 | 2.41 | Azelaic acid | 187.0981 | 188.1049 | $C_9H_{16}O_4$ | 169[-H2O],125[-H2O-COO] |
| M4 | 3.66* | Poricoic acid B-O-sulfate | 563.2817 | 564.2757 | $C_{30}H_{44}O_8S$ | 483[-SO3],337[-C7H14O3] 191[-C19H32O2] |
| C9 | 3.72 | Not identificated 1 | 295.0974 | 296.1048 | C18H16O4 | 277[-H2O],233[-H2O-COO] 205[-H2O-COO-CO] |
| C10 | 3.78 | Not identificated 2 | 295.0967 | 296.1048 | C18H16O4 | 277[-H2O],233[-H2O-COO] 205[-H2O-COO-CO] |
| C11 | 3.90 | Not identificated 3 | 295.0972 | 296.1048 | C18H16O4 | 277[-H2O],233[-H2O-COO] 205[-H2O-COO-CO] |
| M5 | 4.59* | Tumulosic acid-O-glucuronide | 661.3857 | 662.4030 | $C_{37}H_{58}O_{10}$ | 485[-(GluA-H2O)],467[-H2O] 423[-CH2O3] |

| | | | | | | |
|---|---|---|---|---|---|---|
| M6 | 4.61* | 9-Octadecenoic acid-O-glucuronide | 457.2692 | 458.2880 | $C_{24}H_{42}O_8$ | 281[-(GluA-H2O)],255[-C2H6]<br>227[-C2H6-CO] |
| C12 | 5.55 | Polyporenic acid C | 481.3311 | 482.3396 | $C_{31}H_{46}O_4$ | 311[-C9H14O3],153[-C22H32O2] |
| C13 | 5.66 | Poricoic acid B | 483.3123 | 484.3189 | $C_{30}H_{44}O_5$ | 437[-CH2O2],390[-C6H5O]<br>337[-C7H14O3],191[-C19H32O2]<br>126[-C22H29O4] |
| C14 | 5.92 | Tumulosic acid | 485.3633 | 486.3710 | $C_{31}H_{50}O_4$ | 469[-CH4],467[-H2O]<br>423[-CH2O3],337[-C6H12O4] |
| C15 | 6.07 | Poricoic acid A | 497.3269 | 498.3345 | $C_{31}H_{46}O_5$ | 453[-C3H8],435[-CH2O3]<br>397[-C5H8O2],351[-C7H14O3]<br>147[-C22H38O3] |
| C16 | 8.34 | 9-Octadecenoic acid | 281.2484 | 282.2560 | $C_{18}H_{34}O_2$ | 255[-C2H6],227[-C2H6-CO] |

Note：* metabolites

**Table S5**. Identified results of the constituents in rat plasma after oral administration of SQW in positive ion mode.

| NO. | Rt | Identified compounds | [M+H] | mv(Da) | Formula | MS/MS data |
|---|---|---|---|---|---|---|
| C17 | 1.52 | Eugenol methyl ether | 179.0717 | 178.0994 | $C_{11}H_{14}O_2$ | 151[-C2H4],137[-C3H6] |
| M7 | 1.74* | Paeonol-O-glucuronide | 343.1027 | 342.0951 | $C_{15}H_{18}O_9$ | 167[-(GluA-H2O)],175[(GluA-H2O)-H] |
| C18 | 1.93 | Sesterstatin 7 | 445.2965 | 444.2876 | $C_{27}H_{40}O_5$ | 429[-CH4],372[-C4H9O],<br>301[-C8H16O2],117[-C21H43O2] |
| M8 | 2.24* | Alisol C glucuronide | 663.3538 | 662.3666 | $C_{36}H_{54}O_{11}$ | 487[-(GluA-H2O)],469[-(GluA-H2O)-H2O],<br>451-(GluA-H2O)[-2H2O] |
| M9 | 2.76* | Alisol B-23-aceate-O-sulfate | 595.3533 | 594.3226 | $C_{32}H_{50}O_8S$ | 515[-SO3],497[-H2O]<br>365[-H2O-C4H8O-C2H4O2]<br>339[-H2O-C8H14O3] |
| C19 | 3.48 | Hypaconitine | 616.3122 | 615.3043 | $C_{33}H_{45}NO_{10}$ | 584[-CH4O],556[-C2H4O2]<br>524[-C3H8O3],492[-C4H12O4] |
| C20 | 3.68 | 16-oxoalisol A | 505.3535 | 504.3451 | $C_{30}H_{48}O_6$ | 487[-H2O],469[-2H2O]<br>451[-3H2O],415[-C4H10O2] |
| C21 | 3.74 | Paeonol | 167.0718 | 166.0629 | $C_9H_{10}O_3$ | 149[-C2H6],121[-C2H6O] |
| C22 | 5.48 | Alisol B | 473.3624 | 472.3552 | $C_{30}H_{48}O_4$ | 455[-H2O],437-[2H2O],383[-H2O-C4H8O] |
| C23 | 5.50 | Dehydrotrametenolic acid | 455.3433 | 454.3447 | $C_{30}H_{46}O_3$ | 437[-OH] |
| C24 | 5.94 | Dehydroeburicoic acid | 469.3645 | 468.3603 | $C_{31}H_{48}O_3$ | 451[-H2O],313[-C9H16O2] |

Note：* metabolites

**Table S6**. Coefficients of correlation analysis between marker metabolites and absorbed constituents.

| No. | B1 | B2 | B3 | B4 | B5 | B6 | B7 | B8 | B9 | B10 | B11 | B12 | B13 | B14 | B15 | B16 | B17 | B18 | B19 | B20 | B21 | B22 | B23 |
|---|---|---|---|---|---|---|---|---|---|---|---|---|---|---|---|---|---|---|---|---|---|---|---|
| C1 | 0.56 | 0.01 | 0.00 | 0.32 | -0.12 | 0.35 | 0.01 | -0.48 | -0.33 | -0.11 | 0.01 | -0.55 | -0.24 | 0.16 | 0.25 | -0.63 | -0.47 | -0.10 | -0.14 | 0.65 | 0.33 | -0.11 | -0.33 |
| C2 | -0.54 | -0.33 | 0.05 | 0.15 | 0.43 | -0.32 | -0.41 | 0.19 | -0.50 | -0.02 | 0.03 | -0.42 | -0.50 | 0.31 | -0.23 | 0.03 | -0.01 | -0.33 | -0.23 | 0.09 | 0.52 | -0.36 | -0.36 |
| C3 | 0.77 | 0.16 | 0.20 | 0.26 | -0.27 | 0.58 | 0.18 | -0.39 | 0.09 | -0.16 | 0.22 | 0.12 | 0.24 | -0.24 | 0.10 | -0.22 | -0.17 | 0.34 | -0.03 | 0.14 | -0.18 | 0.16 | -0.12 |
| C4 | -0.69 | 0.06 | -0.45 | -0.57 | -0.46 | -0.46 | 0.23 | 0.38 | 0.53 | 0.69 | -0.42 | 0.34 | 0.37 | 0.41 | 0.31 | -0.01 | -0.22 | 0.19 | 0.51 | -0.30 | 0.19 | 0.30 | 0.61 |
| C5 | -0.54 | -0.47 | -0.01 | -0.07 | 0.30 | -0.06 | -0.12 | 0.21 | -0.19 | -0.42 | -0.02 | 0.04 | -0.16 | -0.23 | -0.08 | 0.30 | 0.36 | -0.10 | 0.11 | -0.08 | -0.18 | -0.31 | 0.10 |
| C6 | 0.18 | -0.23 | 0.27 | 0.13 | 0.30 | 0.29 | 0.03 | -0.67 | 0.18 | 0.01 | 0.21 | -0.16 | 0.46 | -0.15 | 0.19 | 0.10 | 0.09 | 0.45 | 0.30 | 0.21 | 0.15 | 0.59 | -0.10 |
| C7 | -0.38 | -0.26 | -0.07 | -0.23 | 0.03 | 0.18 | -0.41 | -0.26 | 0.03 | 0.17 | -0.10 | 0.09 | 0.33 | 0.14 | -0.13 | 0.19 | 0.00 | 0.53 | 0.26 | -0.21 | 0.27 | 0.40 | -0.13 |
| C8 | -0.53 | 0.34 | -0.45 | -0.68 | -0.39 | -0.46 | 0.00 | 0.40 | 0.52 | 0.65 | -0.44 | 0.47 | 0.36 | 0.29 | -0.03 | 0.22 | 0.01 | 0.14 | 0.28 | -0.55 | -0.08 | 0.37 | 0.47 |
| C9 | 0.24 | -0.32 | 0.82 | 0.94 | 0.15 | 0.36 | -0.03 | 0.21 | -0.38 | -0.20 | 0.86 | 0.17 | -0.18 | -0.01 | -0.30 | 0.02 | -0.23 | 0.32 | -0.43 | -0.20 | 0.52 | -0.41 | -0.57 |
| C10 | 0.32 | -0.39 | 0.81 | 0.96 | 0.33 | 0.40 | -0.08 | -0.04 | -0.47 | -0.33 | 0.83 | -0.08 | -0.23 | -0.08 | -0.25 | -0.02 | -0.16 | 0.23 | -0.42 | 0.04 | 0.50 | -0.35 | -0.65 |
| C11 | -0.06 | 0.32 | -0.22 | -0.47 | 0.10 | -0.61 | 0.74 | -0.03 | 0.85 | 0.37 | -0.24 | 0.06 | 0.49 | -0.18 | 0.57 | 0.21 | 0.37 | -0.26 | 0.52 | 0.11 | -0.50 | 0.57 | 0.84 |
| C12 | 0.13 | -0.66 | 0.81 | 0.62 | 0.57 | 0.42 | 0.38 | -0.21 | 0.13 | -0.66 | 0.80 | 0.28 | 0.36 | -0.75 | 0.09 | 0.52 | 0.53 | 0.44 | 0.22 | -0.05 | -0.29 | 0.05 | 0.03 |
| C13 | -0.08 | 0.36 | -0.27 | -0.12 | -0.41 | -0.21 | -0.15 | 0.73 | -0.25 | 0.01 | -0.21 | 0.26 | -0.50 | 0.17 | -0.35 | -0.11 | -0.14 | -0.40 | -0.44 | -0.35 | -0.16 | -0.63 | -0.02 |
| C14 | 0.18 | 0.49 | 0.26 | 0.20 | -0.19 | -0.37 | 0.33 | 0.50 | 0.36 | 0.54 | 0.30 | 0.39 | 0.10 | 0.19 | -0.11 | 0.09 | -0.14 | -0.04 | -0.24 | -0.42 | 0.12 | 0.05 | 0.10 |
| C15 | 0.39 | 0.17 | 0.53 | 0.27 | 0.46 | 0.12 | -0.08 | -0.08 | 0.12 | -0.23 | 0.49 | 0.30 | 0.20 | -0.49 | -0.45 | 0.61 | 0.57 | 0.13 | -0.28 | -0.39 | -0.42 | 0.22 | -0.24 |
| C16 | -0.19 | -0.13 | -0.43 | -0.48 | 0.23 | -0.33 | 0.41 | -0.66 | 0.42 | 0.22 | -0.49 | -0.64 | 0.33 | 0.05 | 0.82 | -0.22 | 0.07 | -0.22 | 0.71 | 0.77 | -0.02 | 0.61 | 0.56 |
| C17 | -0.27 | 0.47 | -0.38 | -0.16 | -0.54 | -0.60 | 0.08 | 0.89 | -0.08 | 0.49 | -0.30 | 0.12 | -0.52 | 0.57 | -0.09 | -0.35 | -0.45 | -0.55 | -0.35 | -0.23 | 0.23 | -0.57 | 0.16 |
| C18 | -0.65 | -0.58 | 0.20 | 0.11 | -0.07 | -0.05 | 0.29 | 0.25 | 0.26 | 0.19 | 0.24 | 0.34 | 0.36 | 0.11 | 0.30 | 0.13 | -0.13 | 0.51 | 0.49 | -0.22 | 0.41 | 0.07 | 0.32 |
| C19 | -0.07 | -0.18 | -0.47 | -0.24 | 0.06 | -0.06 | 0.00 | -0.50 | -0.23 | -0.07 | -0.50 | -0.81 | -0.25 | 0.22 | 0.50 | -0.55 | -0.24 | -0.39 | 0.25 | 0.85 | 0.20 | -0.01 | 0.07 |
| C20 | 0.02 | -0.06 | -0.10 | -0.28 | -0.27 | 0.33 | 0.08 | -0.36 | 0.42 | 0.17 | -0.10 | 0.31 | 0.64 | -0.10 | 0.20 | 0.08 | -0.02 | 0.65 | 0.48 | -0.14 | -0.10 | 0.58 | 0.24 |
| C21 | 0.17 | -0.67 | 0.97 | 0.86 | 0.57 | 0.52 | 0.10 | -0.20 | -0.14 | -0.56 | 0.96 | 0.21 | 0.21 | -0.52 | -0.14 | 0.42 | 0.30 | 0.55 | -0.03 | -0.12 | 0.11 | -0.05 | -0.36 |
| C22 | 0.08 | 0.46 | -0.22 | -0.50 | 0.04 | -0.35 | 0.24 | -0.27 | 0.68 | 0.50 | -0.27 | 0.07 | 0.54 | -0.03 | 0.20 | 0.23 | 0.26 | 0.02 | 0.33 | -0.07 | -0.31 | 0.78 | 0.42 |
| C23 | 0.14 | 0.84 | -0.45 | -0.14 | -0.73 | -0.57 | 0.07 | 0.69 | -0.05 | 0.77 | -0.38 | -0.05 | -0.52 | 0.79 | -0.05 | -0.64 | -0.74 | -0.59 | -0.50 | -0.07 | 0.39 | -0.40 | 0.01 |
| C24 | 0.52 | 0.09 | -0.04 | 0.35 | -0.12 | 0.20 | -0.05 | -0.36 | -0.46 | -0.03 | -0.03 | -0.67 | -0.45 | 0.32 | 0.19 | -0.74 | -0.58 | -0.29 | -0.30 | 0.69 | 0.46 | -0.26 | -0.41 |
| C25 | -0.20 | -0.26 | -0.32 | -0.37 | 0.02 | 0.30 | -0.40 | -0.58 | -0.09 | -0.05 | -0.37 | -0.25 | 0.21 | 0.05 | 0.08 | -0.05 | -0.01 | 0.32 | 0.34 | 0.21 | 0.09 | 0.38 | -0.09 |
| C26 | -0.65 | 0.21 | -0.69 | -0.81 | -0.20 | -0.45 | -0.34 | 0.13 | 0.17 | 0.51 | -0.72 | -0.01 | 0.08 | 0.41 | -0.04 | 0.06 | -0.01 | -0.10 | 0.25 | -0.20 | 0.05 | 0.29 | 0.26 |
| C27 | 0.47 | 0.24 | 0.03 | 0.00 | 0.02 | -0.10 | 0.72 | -0.13 | 0.47 | -0.11 | 0.04 | 0.01 | 0.22 | -0.40 | 0.46 | -0.03 | 0.23 | -0.25 | 0.20 | 0.29 | -0.58 | 0.16 | 0.51 |
| C28 | 0.16 | -0.19 | 0.50 | 0.41 | -0.15 | 0.37 | 0.18 | -0.02 | 0.22 | 0.11 | 0.53 | 0.46 | 0.45 | -0.07 | 0.01 | 0.11 | -0.16 | 0.70 | 0.12 | -0.32 | 0.25 | 0.22 | -0.07 |
| C29 | -0.10 | -0.31 | 0.41 | 0.28 | 0.26 | 0.36 | -0.37 | 0.31 | -0.34 | -0.64 | 0.41 | 0.48 | -0.14 | -0.48 | -0.63 | 0.57 | 0.47 | 0.22 | -0.31 | -0.56 | -0.35 | -0.41 | -0.32 |
| C30 | -0.10 | -0.10 | -0.19 | -0.07 | 0.46 | -0.19 | -0.37 | -0.45 | -0.41 | -0.03 | -0.26 | -0.84 | -0.37 | 0.24 | 0.04 | -0.21 | -0.02 | -0.44 | -0.10 | 0.59 | 0.33 | 0.03 | -0.33 |
| C31 | -0.70 | -0.54 | -0.09 | -0.47 | 0.37 | 0.05 | -0.19 | -0.34 | 0.31 | -0.08 | -0.16 | 0.17 | 0.59 | -0.26 | 0.07 | 0.59 | 0.53 | 0.52 | 0.66 | -0.20 | -0.17 | 0.60 | 0.28 |
| C32 | -0.49 | 0.14 | -0.16 | 0.03 | -0.13 | -0.82 | 0.27 | 0.66 | 0.00 | 0.63 | -0.12 | -0.24 | -0.41 | 0.68 | 0.23 | -0.35 | -0.45 | -0.56 | -0.11 | 0.09 | 0.64 | -0.35 | 0.20 |
| C33 | -0.10 | -0.23 | -0.04 | -0.36 | 0.43 | 0.25 | -0.22 | -0.33 | 0.10 | -0.61 | -0.11 | 0.19 | 0.29 | -0.70 | -0.20 | 0.65 | 0.82 | 0.15 | 0.25 | -0.17 | -0.82 | 0.27 | 0.14 |

Note: B, marker metabolites; C: absorbed constituents.

**Table S7**. Potential targets prediction of the highly correlated ingredients in vivo.

| NO. | Ingredients | Predicting Score | Id | Name | GenBank ID (gene) | GenBank ID (protein) |
|---|---|---|---|---|---|---|
| 1 | Geniposidic acid | 0.60 | 817 | DNA topoisomerase 2-alpha | J04088 | 292830 |
| 2 | Azelaic acid | 0.81 | 6105 | 3-oxo-5-beta-steroid 4-dehydrogenase | Z28339 | 431857 |
| 3 | Azelaic acid | 0.81 | 279 | Tyrosinase | M27160 | 340037 |
| 4 | Tumulosic acid-O-glucuronide | 0.61 | 737 | Mineralocorticoid receptor | M16801 | 307166 |
| 5 | Tumulosic acid-O-glucuronide | 0.60 | 871 | Glucocorticoid receptor | X03225 | 31680 |
| 6 | Poricoic acid B | 0.60 | 737 | Mineralocorticoid receptor | M16801 | 307166 |
| 7 | Poricoic acid A | 0.60 | 737 | Mineralocorticoid receptor | M16801 | 307166 |
| 8 | 9-Octadecenoic acid | 0.73 | 1395 | Sodium/calcium exchanger 1 | M91368 | 180673 |
| 9 | 9-Octadecenoic acid | 0.70 | 3993 | Delta-6 fatty acid desaturase | AF126799 | 4406528 |
| 10 | Eugenol methyl ether | 0.69 | 556 | Alpha-1A adrenergic receptor | D25235 | 433201 |
| 11 | Eugenol methyl ether | 0.66 | 540 | Sodium-dependent noradrenaline transporter | M65105 | 189258 |
| 12 | Eugenol methyl ether | 0.65 | 193 | Beta-1 adrenergic receptor | J03019 | 178200 |
| 13 | Alisol C glucuronide | 0.65 | 871 | Glucocorticoid receptor | X03225 | 31680 |
| 14 | Alisol C glucuronide | 0.64 | 737 | Mineralocorticoid receptor | M16801 | 307166 |
| 15 | Hypaconitine | 0.63 | 1588 | Multidrug resistance protein 1 | M14758 | 307180 |
| 16 | Paeonol | 0.66 | 828 | Phenylalanine-4-hydroxylase | K03020 | 189937 |
| 17 | Paeonol | 0.63 | 275 | Arachidonate 5-lipoxygenase | J03600 | 187193 |
| 18 | Paeonol | 0.63 | 3949 | Prolyl 3-hydroxylase 2 | AJ430351 | 27526730 |

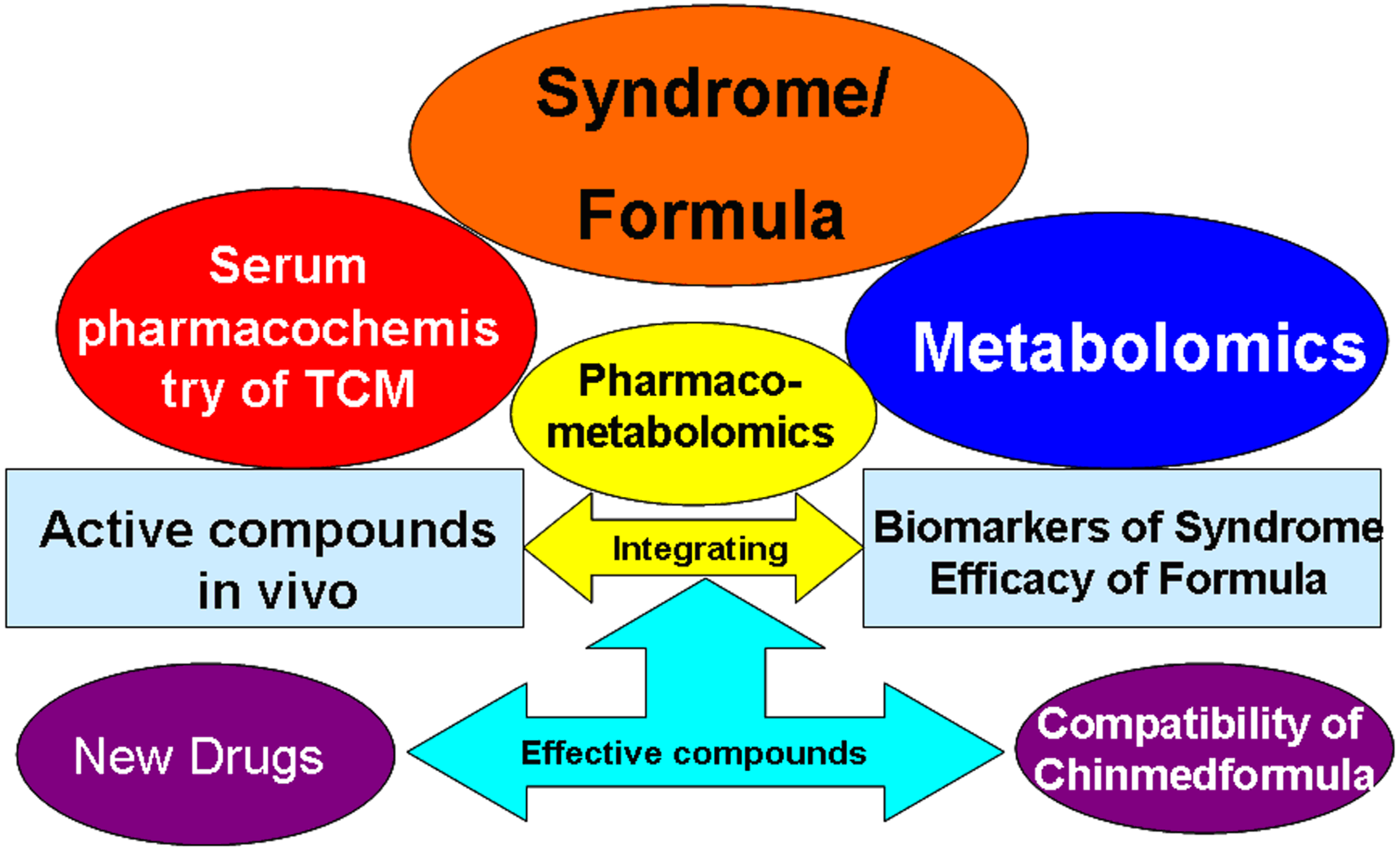


**Figure S1** Flow chart: the research strategy underpinning chinmedomics.
